# Clinical and radiographical results of labral reconstruction

**DOI:** 10.1093/jhps/hnv062

**Published:** 2015-11-04

**Authors:** Roland S. Camenzind, Isabelle Steurer-Dober, Martin Beck

**Affiliations:** 1. Clinic of Orthopaedic Surgery, Luzerner Kantonsspital, Spitalstrasse 6003 Lucerne, Switzerland; 2. Department of Radiology, Luzerner Kantonsspital, Spitalstrasse, 6000 Lucerne, Switzerland.

## Abstract

Treatment of femoroacetabular impingement (FAI) includes correction of underlying bony deformities. Labrum preservation is recommended whenever possible. In hips, where the labrum is missing or damaged beyond preservation, labral reconstruction is an option to restore labral seal. Between 2008 and 2011, 84 hips underwent treatment for FAI by means of a surgical hip dislocation. In 13 of these hips (11 patients), the severely damaged or missing labrum was reconstructed with ligamentum capitis femoris. Pre- and postoperative radiographic and clinical data were analysed with a mean follow-up of 38 months (range: 19–65 months). Clinical outcome was determined with Oxford hip score (OHS) and overall satisfaction, rest and load pain with a visual analogue scale (VAS; 0–100). Clinical outcome was compared with a control group where labral refixation was performed. Mean OHS improved significantly (*P* ≤ 0.001) from 29 (SD 8) to 44 (SD 4). Overall satisfaction with the hip increased significantly (*P* = 0.002) from 44 (SD 35) to 87 (SD 15). Mean VAS for rest pain decreased significantly (*P* = 0.0004) from 45 (SD 35) to 5 (SD 7) as well as for load pain (*P* = 0.0007) from 59 (SD 26) to 16 (SD 19). There were no significant differences between the two groups. Reconstruction of the acetabular labrum with ligamentum capitis femoris yields good clinical results. Technical superiority of open labral reconstruction may explain the unexpected, excellent outcome.

## INTRODUCTION

Femoroacetabular impingement (FAI) is an important cause of groin pain, cartilage and labrum damage and a risk factor for the development of osteoarthritis (OA) of the hip [1–4]. FAI is caused by pathological deformities of either the femoral head or acetabulum that lead to a pathological conflict between acetabular rim and femoral neck and are responsible for the development of damage of the acetabular rim including the labrum and cartilage [2, 5].

The labrum plays an important role in hip joint stability and maintenance of articular cartilage health. It increases the effective surface area of the hip joint, deepens the acetabular socket and serves as a fluid seal [6–8]. It contributes to the stability of the hip joint by its valve effect and structure [9, 10]. In addition, because the labrum adds resistance to the flow path for synovial fluid expression, cartilage indentation is significantly quicker without the labrum. Both of these mechanisms are dependent on the fit of the labrum against the femoral head [11]. Disruption of the labral seal could have adverse effects on joint lubrication and friction of the cartilage surfaces leading to its premature degeneration [12, 13].

The current literature supports the role of preservation of the native labrum whenever possible resulting in improved clinical and radiological outcome [14–16]. Timing and necessity for labral reconstruction in the presence of a deficient or nonusable labrum remains controversial.

The aim of this study was to review the results of labral reconstruction with ligamentum capitis femoris during surgical hip dislocation in situations where the labrum was missing or beyond salvage and to assess anatomical integrity of the reconstructed labrum with magnetic resonance (MR)-arthrography.

## MATERIALS AND METHODS

Between January 2008 and October 2011, 84 hips (79 patients) underwent surgical hip dislocation for treatment of FAI, of which in 14 hips (12 patients, 16%) the labrum was reconstructed. Inclusion criteria were all patients with segmental or circumferential reconstruction of the labrum during the study period with a minimum follow-up of 2 years. Exclusion criteria were OA > Tönnis Grade 1. One hip with OA Grade 2 and severe cartilage damage was excluded from the study, leaving 13 hips in 11 patients. As a control a group of patients was selected who had surgery during the same time period with labral reattachment. The group consisted of 14 hips in 11 patients. The hips were selected based on following parameters: age < 40 years, no radiographically visible degenerative changes (OA Tönnis = 0) and no previous operations.

Radiographic and clinical examinations were done preoperatively, at 6 weeks and 1 year postoperatively. In addition, a questionnaire was sent to the patients at the time of last follow-up. Radiographic analysis was carried out on a/p pelvic and lateral cross table radiographs. Joint degeneration was graded using the Tönnis classification. Radiographic analysis included measurement of the acetabular index angle, lateral centre edge (LCE) angle, alpha angle and the presence of acetabular retroversion. Acetabular retroversion was quantified with the retroversion index [17]. Based on the presence of a cam deformity and the depth and orientation of the acetabulum, FAI was classified as cam, pincer or mixed. The presence of an asphericity angle larger than 55° was considered a cam-FAI. A pincer-FAI was present when the LCE angle was larger than 35° and the acetabular retroversion index was more than 33% [17]. The demographic data of both groups are summarized in Table I.

**Table I.** Details of the patients in the two groups: patient demographics, radiographic findings and follow-up

	Total	Reconstruction	Control group
Hips (patients)	27 (22)	13 (11)	14 (11)
Left/right side	12/15	7/6	5/9
Female/male	8/19	5/8	3/11
Mean age (range) in years	30	36 (20–51)	25 (16–40)
Previous surgery	4	4	0
CAM-FAI (%)		4 (31)	11 (79)
Pincer-FAI (%)		6 (46)	2 (14)
Combined-FAI (%)		3 (23)	1 (7)
Mean Tönnis score	0.24	0.38	0.11
Mean (range) follow-up in months	40	38 (19–65)	42 (22–58)

Function of the hip was assessed with the Oxford hip score (OHS) [18, 19] and visual analogue scale (VAS) for satisfaction, pain at rest and load pain. The OHS ranges from a minimum of 0 to a maximum of 48 points [18]. A score equal or higher than 40 indicates an acceptable result [19]. An absolute increase of OHS equal or higher than 6 points is considered the cut-off value for a successful outcome [20]. Graduation of the results was performed as follows: OHS ≥ 42 was considered an excellent, OHS 34–41 a good and OHS 27–33 a fair result [21]. The global satisfaction with the operated hip and pain scores were assessed with a VAS ranging from a minimum of 0 to a maximum of 100 points.

Anatomical integrity of labral reconstruction and cartilage was controlled with MR-arthrography in 11 hips after a mean of 30 months (11–57 month) after surgery. Signal intensity of labral reconstruction was assessed, compared with the native, intact labrum and classified as hyper- or isointense. Cross-section of the reconstruction was calculated: width * height/2 (Fig. 4). The criteria for morphological integrity that we used to diagnose a tear were (i) high signal-intensity line to the surface of the reconstruction with or without formation of a paralabral cyst, (ii) completely torn or (iii) absent labrum. MR images were reviewed in a consensus reading by one fellowship trained musculosceletal radiologist (I.S.-D.) with 7 years and a senior orthopaedic surgeon (M.B.) with 14 years of experience in interpreting hip magnetic resonance imaging (MRI). Analysis was performed using the local PACS (Merlin PACS, Phönix-PACS, Freiburg, Germany) in a random order and the readers were blinded to the clinical outcome data.

### Surgical technique

**Fig. 1.**
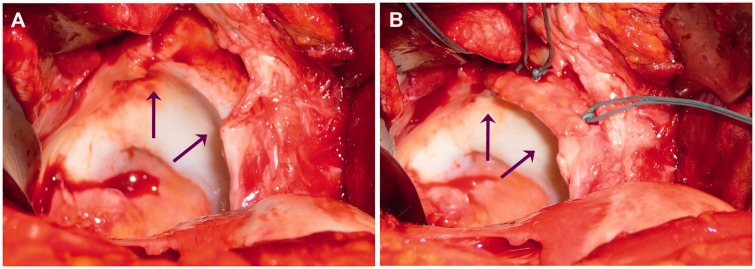
Intraoperative photograph while performing a reconstruction of acetabular labrum. (**A**) Degenerated parts of the labrum were debrided and the acetabular rim was trimmed (area between the two arrows). (**B**) The prepared ligament was attached with three bone anchors using simple stitches.

Surgical hip dislocation was performed in all hips as described by Ganz *et al.* [22] with a stepped trochanter osteotomy [23, 24]. The ligamentum capitis femoris was cut with strong curved scissors close to the transverse ligament. Deep dissection and correction of bone abnormalities were done as described before [25]. Intraoperative findings and corrections were recorded. In areas where the labrum was degenerated, ossified or absent the acetabular rim was trimmed or debrided, depending on the amount of necessary resection, until the bone was bleeding. The ligamentum capitis femoris was harvested cutting it close to fovea capitis femoris. The synovium was removed until only the longitudinal fibres remained and a strand was prepared with a diameter of about 4–5 mm. Depending on the length of the reconstruction, the ligament was divided with an incomplete simple or z-shaped cut and stretched. The prepared ligament was attached with bone anchors (TWINFIX Ti 2.8 Suture Anchor; Smith & Nephew, Andover, MA, USA) using simple stitches (Fig. 1). In one case where the ligamentum capitis femoris could not be used, a strip of fascia lata was tubulized and reattached as described above. After reduction of the femoral head the suction seal was tested and reattachment of the reconstructed labrum improved if necessary. In such situation the bone anchor was removed, a new suture threaded in to the anchor, which was then reused. The capsule was closed loosely and the trochanter reattached with two screws.

### Postoperative care and follow-up

The postoperative care followed the same principles as for a ‘normal’ surgical dislocation. The wound was inspected on the second postoperative day and the sutures were removed after 10 days. Partial weight-bearing with 15 kg was allowed for 4 weeks, followed by gradual increase of weight bearing. Passive continuous motion was started on the first day after surgery and continued for 4 weeks three times a day for 30 min. When radiological evidence of union of the osteotomy of the greater trochanter was seen, usually 6 weeks after surgery, full weight-bearing was allowed.

### Statistical analysis

For all statistical analyses, we used SPSS 19.0 software (SPSS, Chicago, IL, USA). Normality of data was ascertained by Q–Q plots. Student’s paired *t*-tests were used to compare data from pre-intervention with data from post-intervention. And student’s unpaired *t*-tests were used to compare absolute changes from preoperative to postoperative state. Statistical significance was set at *P* < 0.05.

Approval for the study was given by the local Ethics Committee and informed consent was obtained from all patients.

## RESULTS

No wound healing problems or infections were observed in any of the hips. All 13 hips had a minimum follow-up of 19 months. There were 11 patients (six men, five women) with a mean age of 36 years (range, 20–51 years) and with mean follow-up of 38 months (19–65 months). Demographics details of the two groups are shown in Table I.

An osteochondroplasty was done in 12 of 13 hips. Resection of the acetabular rim was carried out in all hips with reconstruction of the labrum. A detailed overview of the intraoperative findings and corrections is given in Table II.

**Table II.** Intraoperative findings: location of femoral and acetabular cartilage, labral damage, location of rim resection, labral reconstruction/refixation and offset correction

No.	FAI	Femoral cartilage	Acetabular cartilage	Labral damage	Location of rim resection	Labral reconstruction	Offset correction
1	CAM	No damage	Lesion 10′–1′	1′–3′	12′–4′	1′–3′	Yes
2	CAM	No damage	No damage	1′–3′	11′–4′	1′–3′	Yes
3	CAM	No damage	Lesion 12′	2′–3′	10′–4′	1′–3′	Yes
4	Pincer	No damage	Lesion 12′	12′–3′	9′–4′	12′–3′	Yes
5	Combined	Osteophytes	No damage	9′–5′	3′–5′	3′–5′	Yes
6	Combined	No damage	12′–2′	12′–5′	12′–5′	12′–4′	Yes
7	CAM	Damaged	No damage	12′–4′	11′–5′	12′–3′	Yes
8	Pincer	No damage	No damage	10′–2′	10′–5′	10′–2′	No
9	Pincer	5 × 8 mm lesion	No damage	1′–4′	8′–4′	1′–4′	Yes
10	Pincer	No damage	10′–3′	8′–4′	8′–4′	8′–4′	Yes
11	Pincer	No damage	No damage	1′–2′	10′–4′	1′–2′	Yes
12	Pincer	No damage	Contre coup	10′–12′	7′–4′	10–12′	Yes
13	Combined	No damage	No damage	7′–8′	4′–11′	7′–8′	Yes

The location of labral damage and reconstruction is presented using a clock face system. For better comparison, left hips were converted to right hips.

**Fig. 2.**
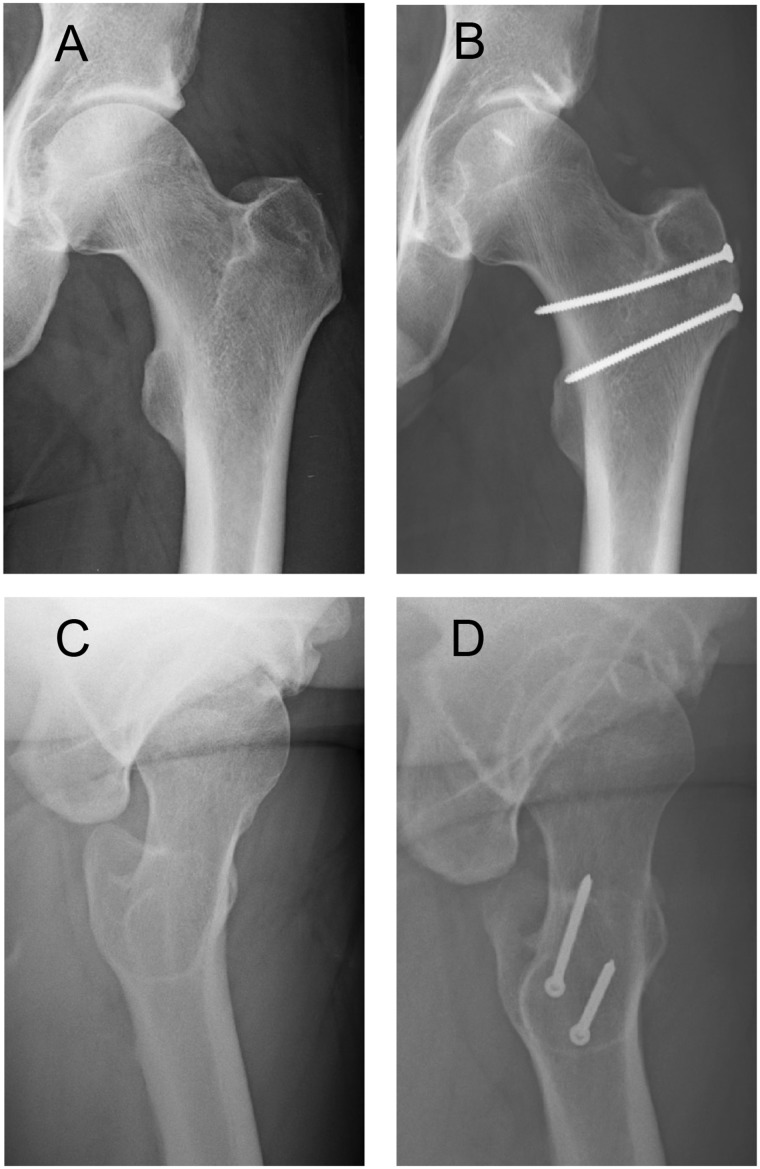
Pre- (**A, C**) and postoperative (**B, D**) X-ray of the left hip of Patient Number 2. (A) Preoperative X-ray in a/p pelvic view with an LCE of 39°, and (B) corrected LCE of 31° postoperatively. (C) Alpha angle of 62° preoperatively was corrected to (D) 44° postoperatively in the lateral cross table radiograph. Acetabular labrum was fixed with four bone anchors from.

The LCE was corrected from a mean of 36° (range: 26–50) preoperatively to a mean of 26° (range: 19–31). The alpha angle was corrected from a mean of 62° (range: 47–75) to a mean of 45° (range: 40–55). Details of clinical results and radiological changes are shown in Table III. Figure 2 illustrates an example of pre- and postoperative X-rays.

**Table III.** Comparison of pre- and postoperative clinical scores, pain values and radiological changes

No.	Tönnis	OHS		Satisfaction		Rest pain		Load pain		Lateral centre edge		Alpha angle	
	grade	Preop	Postop	Preop	Postop	Preop	Postop	Preop	Postop	Preop	Postop	Preop	Postop
1	0	36	48	95	100	10	0	40	0	26	19	71	41
2	0–1	35	46	20	90	70	0	40	10	39	31	62	44
3	0	35	41	70	85	20	10	60	20	36	26	60	49
4	0	24	46	80	100	30	0	30	0	32	23	61	44
5	1	29	44	30	75	40	5	60	20	36	26	71	45
6	1	47	48	100	90	0	0	10	15	41	28	61	48
7	0	24	36	60	50	20	10	40	70	29	25	70	43
8	0	22	43	0	100	80	0	90	10	38	30	47	40
9	0	20	41	0	90	80	20	80	20	40	23	62	40
10	1	24	45	30	80	50	15	85	20	43	30	75	43
11	0–1	32	48	35	100	65	0	75	0	32	27	50	45
12	0	15	46	5	100	85	0	95	0	50	30	61	55
13	1	36	37	40	70	30	10	60	25	34	28	60	48

**Fig. 3.**
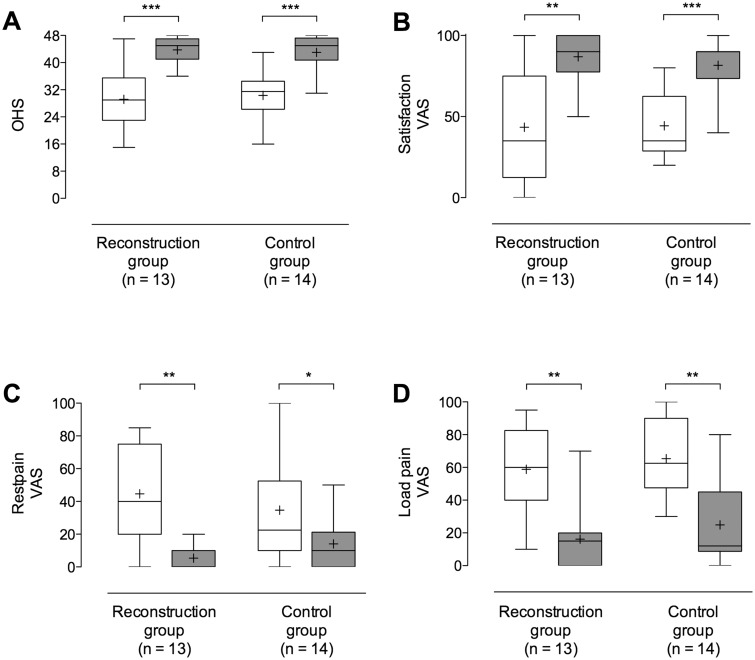
Comparison of clinical outcome scores between reconstruction and control group represented with boxplots. White bars show preoperative and grey bars postoperative values. Comparison of pre- and postoperative values for (**A**) Oxford Hip score, (**B**) satisfaction, (**C**) pain at rest and (**D**) load. Mean value is represented with ‘+’ and whiskers indicated minimum to maximum values.

All 11 patients (13 hips) reported improvement of their symptoms (Fig. 3A–D). The mean OHS improved significantly (*P* < 0.001) from 29 (SD 9) to 44 (SD 4). The mean change of OHS was 15 (SD 9). Eleven out of 13 (85%) hips had an improvement of the OHS ≥ 6. Eleven out of 13 hips (85 %) had an OHS ≥ 40. One out of three hips with an OHS ≤ 40 had arthroscopic resection of the labrum 24 months before reconstruction. Ten hips (77%) had an excellent result and three (23%) a good result. Patient satisfaction improved significantly (*P* = 0.002) from preoperatively 44 (SD 35) to 87 (SD 15) at last follow-up. VAS for pain at rest could be improved from a mean of 45 (SD 29) to 5 (SD 7) (*P* = 0.0004) and for load pain from 59 (SD 26) to 16 (SD 19) (*P* = 0.0007).

**Fig. 4.**
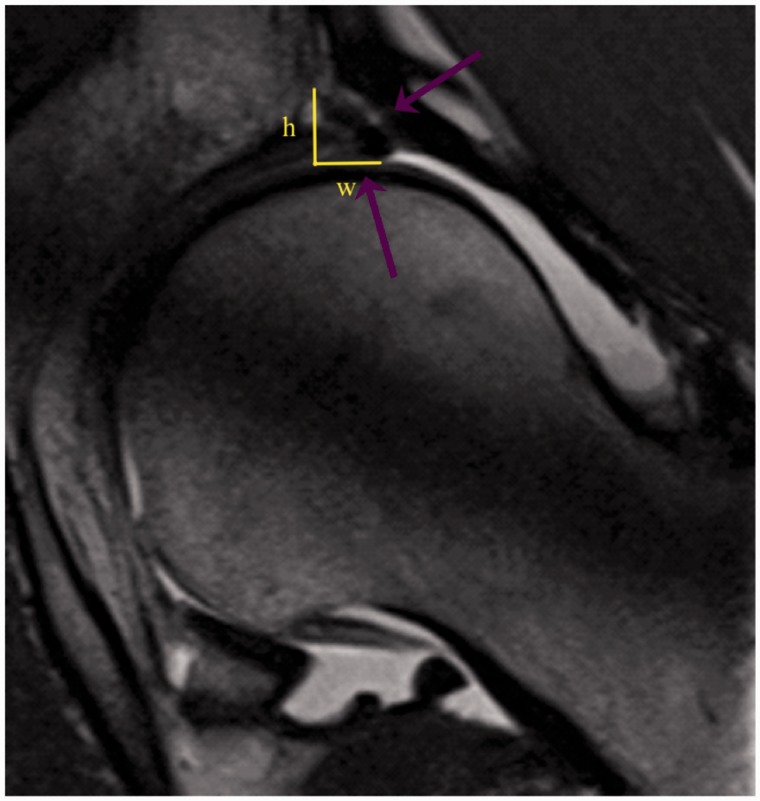
Postoperative MR-arthrography. The two arrows show the reconstructed acetabular labrum between two bone anchors, which is well-attached to the acetabular rim. There is no tear or fluid between the ligament and bone detectable. Cross-section of the reconstruction was calculated width (w) * height (h)/2.

In the control group, the mean OHS improved significantly (*P* < 0.001) from 30 (SD 8) to 44 (SD 5) after surgery. The mean change of OHS was 14 (SD 10). Nine out of 14 hips (64%) had an improvement of OHS ≥ 6. Eleven out of 14 hips (79%) had an OHS ≥ 40 postoperatively. Eleven patients (79%) had an excellent result, 2 (14%) a good and 1 (7%) a fair result. There were no previous operations concerning the hip in this group. There was no significant difference in all measured clinical scores between the two groups (*P* > 0.05).

MR-arthrography demonstrated in all hips the presence of a reconstructed labrum at the acetabular rim (Fig. 4, Table IV). The majority (74%) of labral reconstruction was isointense compared with the native labrum. The size of the reconstructed labrum averaged 18 mm^2^. In 4 of 11 hips of the reconstruction group following pathological structures in the labrum were detected: 2× ganglion, 1× tear, 1× adhesion. No correlation between detected pathological structure and clinical outcome could be found (Table IV).

**Table IV.** MR-arthrography, reconstruction size, signal intensity, labral pathology, pathology type and clinical scores

Patient	Ø mm^2^	Isointensity (%)^a^	Pathology (%)^b^	Pathology type	OHS	Satisfaction
2	15.5	67	0	None	46	90
3	12.0	100	0	None	41	85
4	28.5	100	25	Scar	46	100
5	16.5	0	0	None	44	75
6	6.5	67	0	None	48	90
7	13.5	75	40	Ganglion	36	50
9	35.5	67	0	None	43	90
10	12.0	67	0	None	45	80
11	11.5	100	100	Ganglion	48	100
12	18.5	67	0	None	46	100
13	26.5	100	100	Tear	37	70

^a^% of the labral reconstruction shows isoitensity with native acetabular labrum.

^b^% of the labral reconstruction a pathological structure was detected.

One complication was observed: although a step osteotomy of the trochanter was routinely performed to provide better stability of trochanter fixation, one patient had a nonunion of the osteotomy that was successfully revised after 6 months. Another patient complained about pain at the greater trochanter. Removal of the screws resolved the problem and his OHS improved from 30 to 41. We observed no complication associated with the labral reconstruction itself.

## DISCUSSION

The aim of surgical treatment of FAI, be it open or arthroscopic, is to treat the pathology leading to FAI and its sequelae [25]. It was shown that a resected labrum does not regrow [26]. The current literature supports the role of preservation of the native labrum whenever possible resulting in improved clinical and radiological outcome [14–16]. Timing and necessity for labral reconstruction in the presence of a deficient or non-usable labrum remains controversial. The rationale is that reconstruction of the absent or insufficient labrum has the potential of restoring hip stability while recreating labral sealing properties and secondary to that superior clinical results.

Surgical dislocation with correction of FAI can be done safely and yields good to excellent results in patients with early degenerative changes not exceeding Tönnis Grade 1 OA [27–29]. More often seen in pincer-FAI, the labrum shows various stages of degeneration, which makes preservation of the remaining labral tissue difficult or impossible. In situations where no labral tissue is left for preservation, it seems legitimate to reconstruct the labrum in order to restore sealing function. Previous publications report reasonable outcome using the ligamentum capitis femoris [30, 31], fascia latae [32] or gracilis autograft [33]. A recent review revealed improved outcome after labral reconstruction, the main indication being a deficient labrum after previous surgical removal or irreparable tears in young patients [34]. Arthroscopic reconstruction of the labrum with gracilis autograft showed superior results than refixation alone [33].

The present study shows that labral reconstruction with the described technique leads to good clinical results an increase of OHS from an average of 29–44. Hips in the reconstruction group have increased risks for unsatisfactory outcome including higher OA grade, older age [28], female gender and pincer-FAI [34]. Clinical outcome was very good and comparable to a control group where the native labrum could be preserved. Although the control group includes better risks (male gender, younger age, higher incidence of cam-FAI and less joint degeneration), the clinical outcome was not significantly better. The good results of the reconstruction group may be attributed to the reestablished labral seal. Also the group with labral reconstruction showed a higher overall satisfaction, lower load pain and lower pain at rest.

The results are in agreement with the literature for joint preserving hip surgery [28] and seem to be superior to other studies on open [30, 31] or arthroscopic labral reconstructions [32, 35]. The good outcome may be related to the open technique with unrestricted approach to the pathology and the possibility to restore the suction seal and test it by re-dislocating the hip with checking the presence of the vacuum sound. Also the correction of FAI can be always carried out very precisely, leaving no remaining FAI. For open labral reconstruction, the ligamentum capitis femoris is the ideal graft. It is easy to harvest without additional morbidity, and its longitudinal fibres yield excellent tensile strength [36] and make it therefore suitable to replace the labrum. The open procedure allows for a high precision in graft placement and docking to the remaining labrum can easily performed by sutures. One unparalleled advantage of open surgery is the possibility to assess suction seal and to improve fixation and/or placement without restrictions. The use of threaded bone anchors gives the possibility to remove them and furnish with them new sutures to improve the fixation of the labral reconstruction until an optimal position is found. The technical superiority of open labral reconstruction may also explain the excellent outcome with this technique.

MR-arthrography showed for the majority of the reconstructed labra structural and isointensity with the adjacent native labrum. In some patients, pathological structures were found. These findings had no correlations with the clinical outcome. Further studies to correlate the MR findings with clinical outcome are necessary.

Weaknesses of this study include its retrospective nature and comparison to a equally sized control group. As control we used a subset of patients where a good to excellent results is expected. This includes hips of male patients without degenerative changes, mainly presenting with a cam deformity and anterior overcoverage, which are treated with surgical dislocation, offset correction and rim resection with preservation of the labrum. These patients have a postoperative mean OHS of to 43 (SD 6), which improved significantly (*P* < 0.001) from 30 (SD 8) before surgery. This is almost equal to the labral reconstructions and shows the efficacy of the reconstruction technique.

Postoperative MRI was performed on three different MR scanners (1.5 and 3.0 T). However, we believe that the strength of the magnet has no influence on detection of tears or signal intensity abnormalities of the labrum reconstruction. There are studies supporting the use of 1.5-T MR-arthrography for hip imaging with good accuracy and reproducibility for the detection of labral abnormalities [37].

## CONCLUSIONS

Labral reconstruction with ligamentum capitis femoris during surgical hip dislocation is safe and yields good to excellent results in the majority of patients. The results are superior to those reported in the literature, where the damaged labrum was resected and also to some reconstructive arthroscopic techniques. It has to be considered as a valuable option with no adverse effects in those hips where the native labrum cannot be preserved.

## Ethical Review Committee Statement

Approval for the study was given by the local Ethics Committee.
